# Effect of the COVID-19 pandemic on HIV, malaria and tuberculosis indicators in Togo: an interrupted time series analysis

**DOI:** 10.1136/bmjgh-2023-013679

**Published:** 2024-04-03

**Authors:** Yao Rodion Konu, Fall Dogo, Claver Anoumou Dagnra, Tinah Atcha-Oubou, Fifonsi Adjidossi Gbeasor-Komlanvi, Kossivi Agbelenko Afanvi, Fatoumata Binta Tidiane Diallo, Mahmoud Teouri, Moustafa Mijiyawa, Didier Koumavi Ekouevi

**Affiliations:** 1 Département de Santé Publique, Université de Lomé, Lome, Togo; 2 Centre Africain de Recherche en Epidemiologie et en Santé Publique (CARESP), Lomé, Togo; 3 Global Health in the Global South (Inserm UMR 1219, IRD EMR 271), Bordeaux Population Health, Université de Bordeaux, Bordeaux, France; 4 Programme national de lutte contre la tuberculose (PNLT), Lomé, Togo; 5 Programme national de lutte contre le sida, les hépatites virales et les infections sexuellement transmissibles (PNLS-HV-IST), Lomé, Togo; 6 Programme national de lutte contre le paludisme (PNLP), Lomé, Togo; 7 World Health Organization (WHO) Togo Office, Lomé, Togo; 8 Direction du système national d’information sanitaire et l’informatique (DSNSI), Lomé, Togo; 9 Ministère de la Santé, de l’Hygiène Publique et de l'Accès Universel aux Soins, Lomé, Togo

**Keywords:** COVID-19, HIV, Tuberculosis, Malaria, Other study design

## Abstract

**Background:**

Limited data are available on the effects of the COVID-19 pandemic on health-related indicators in sub-Saharan Africa. This study aimed to estimate the effect of the COVID-19 pandemic on nine indicators of HIV, malaria and tuberculosis (TB) in Togo.

**Methods:**

For this interrupted time series analysis, national health information system data from January 2019 to December 2021 and TB programmatic data from the first quarter of 2018 to the fourth quarter of 2022 were analysed. Nine indicators were included. We used Poisson segmented regression to estimate the immediate impact of the pandemic and per-pandemic period trends through incidence rate ratios (IRRs) with 95% CIs.

**Results:**

Overall, there was a decrease in six of the nine indicators, ranging from 19.3% (IRR 0.807, 95% CI 0.682 to 0.955, p=0.024) for the hospitalisation of patients for malaria to 36.9% (IRR 0.631, 95% CI 0.457 to 0.871, p=0.013) for TB diagnosis by *Mycobacterium tuberculosis* Xpert immediately after the declaration of the COVID-19 pandemic. A comparison of the observed and predicted trends showed that the trend remained constant between the prepandemic and pandemic periods of COVID-19 for all malaria indicators. A significant downward monthly trend was observed in antiretroviral therapy initiation (IRR 0.909, 95% CI 0.892 to 0.926, p<0.001) and positive TB microscopy (IRR 0.919, 95% CI 0.880 to 0.960, p=0.002).

**Conclusion:**

HIV, malaria and TB services were generally maintained over time in Togo despite the COVID-19 pandemic. However, given the decline in levels immediately after the onset of the pandemic, there is an urgent need to improve the preparedness of the healthcare system.

**WHAT IS ALREADY KNOWN ABOUT THIS TOPIC**

- Health service disruptions following epidemics in general and specifically the COVID-19 pandemic have been reported in various settings worldwide.
- Assessing the effect of the pandemic on essential health services using routine data is necessary to inform health system responses; however, little evidence exists on the magnitude of disruptions in sub-Saharan African countries.

**WHAT DOES THIS STUDY ADD**

- Based on data from Togo's health information system, a significant decrease in six of the nine indicators was observed immediately after the start of the COVID-19 pandemic. For instance, hospitalization of patients for malaria fell by 19.3%, while diagnosis of tuberculosis by MTB Xpert decreased by 36.9%.
- We found a significant decrease in the levels of all malaria indicators immediately after the onset of the COVID-19 pandemic, followed by a constant trend during the following months.
- The immediate decrease in HIV PCR testing among infants at 6 weeks and in the number of TB case notifications was followed by a gradual improvement in the following months during the pandemic period.

**HOW MIGHT THIS STUDY AFFECT RESEARCH, PRACTICE, OR POLICY**

- Although the number of services appears to have been maintained over time, we did not study the effect of the COVID-19 pandemic on quality of care.
- Future research could fill this gap to better understand why some services were maintained despite the outbreak of COVID-19 and to learn from best practices in preparedness of future pandemics.

## Background

Health system resilience is defined as the ability of a system to prepare for, manage and learn from shocks.^1^ Especially during health crises such as the COVID-19 pandemic, there is an acute need for robust and resilient health systems. These systems should simultaneously fulfil two functions: responding to the crisis and maintaining the provision of essential health services.^2^ In sub-Saharan Africa, care for HIV, malaria and tuberculosis (TB) is essential. Indeed, the continent bears the brunt of the burden of these diseases, also known as the ‘big three diseases’.^3 4^

As of 30 June 2023, in the WHO African region, 9.5 million cases of COVID-19 (175 369 deaths) were recorded.^5^ This represented approximately 1.2% of the total number of cases worldwide.^5^ Some authors have suggested that the indirect effects of the COVID-19 pandemic could severely intensify the burden of HIV infection, malaria and TB and could reverse decades of progress in improving health outcomes in Africa.^6 7^ Furthermore, modelling studies have predicted that in high-burden settings, essential healthcare service disruptions may have generated up to 10%, 20% and 36% excess deaths due to HIV, malaria and TB, respectively, than in the absence of the COVID-19 pandemic.^8^

Several studies have described the magnitude of the decline in or disruption of treatment for the big three diseases during the COVID-19 pandemic.^8–11^ Many studies have covered only the first few months of the pandemic, small geographical areas or a limited number of health facilities. Several reasons have been proposed to explain the decline in healthcare use during the pandemic. These include the public’s fear of contagion, the cancellation of non-COVID-19-related care, health workforce-related disruptions (eg, clinical staff redeployment to provide COVID-19 relief or insufficient human health resources to provide services), barriers (eg, curfews, transportation closures and stay-at-home orders) and constraints (eg, financial difficulties) imposed by containment policies.^11^

In Togo, the first case of COVID-19 was reported on 6 March 2020; by 29 June 2023, 39 504 cases had been confirmed, and 291 deaths had been reported.^12^ To date, the country has experienced three epidemic waves, with the majority of cases reported in the Grand Lomé health region around the capital.^12^ In response to the pandemic, the Togolese government promoted barriers and general measures (curfews, closures of certain towns, transport restrictions, etc). The public health sector reoriented several hospitals and care units, reassigned health personnel and diverted medical equipment and supplies to the care of COVID-19 patients.^12^

Little evidence is available on the effect of the COVID-19 pandemic on health-related indicators and outcomes in sub-Saharan Africa, specifically in Togo. Assessing the effect of the pandemic on essential health services using routine data is necessary to inform health system responses. This approach has the potential to accelerate the knowledge of local stakeholders and countries about the effectiveness of health system adaptations to maintain essential health services during the pandemic.^13^ To our knowledge, only one study has examined the early indirect effects of the pandemic on income variation and household food security.^14^ Thus, there is currently limited evidence on the effects of the pandemic on health service provision related to the big three diseases in Togo.

The aim of this study was to estimate the effect of the COVID-19 pandemic on a selection of indicators of HIV infection, malaria and TB. The findings from this work can generate insights into the indirect effects of the COVID-19 pandemic and inform current and future pandemic preparedness.

## Methods

### Study design

We performed single (uncontrolled) interrupted time series analysis (ITSA) of the data before and after the onset of the COVID-19 pandemic. We used routine monthly health service delivery data for the 36-month period from 1 January 2019 to 31 December 2021, for indicators related to HIV and malaria. The TB data were quarterly data covering the period from quarter 1 2018 to quarter 4 2022 (20 quarters).

### Setting and analysis time points

Togo is a West African country of 56 785 km^2^.^15^ The population was 8.09 million in 2023, 51.3% of which were women.^16^ Most of the population is young (median age of 18.9 years) and lives in rural areas (57.1%).^16^ The health system divides the country into 6 health regions and 39 health districts. The first case of COVID-19 in Togo was reported in March 2020, and mitigation measures were established by the end of the month.^12^ Therefore, for our analysis, we defined (1) the prepandemic (baseline) period as 15 months (9 quarters for TB), from January 2019 to March 2020 (Q1 2018 to Q1 2020 for TB) and (2). We described the pandemic period as 21 months (11 quarters for TB), from April to December 2021 (Q2 2020 to Q4 2022 for TB).

### Data sources and management

For the present analysis, we used HIV and malaria data, which are systematically recorded in the District Health Information System V.2.39.2.1 (DHIS 2). These datasets cover services provided nationwide by a total of 2152 health facilities legally recognised by the Ministry of Health. TB indicators are collected by TB-treating health facilities that report to the national TB control programme. Data from all 90 TB-treating health facilities as of 2022 were used. All the data cover both the public and private subsectors of the country’s health system and are primarily collected daily and subsequently aggregated (either monthly or quarterly) before being recorded in the DHIS 2 or reported to the TB control programme. These data are regularly cleaned and validated by the Ministry of Health division in charge of data monitoring.

We used aggregated data representing the number of events per month for the HIV and malaria data and per quarter for the TB data. Therefore, we had no missing data or outliers in our dataset. We assumed that field data collection was, therefore, stable over time. Indeed, during the COVID-19-related restrictions, healthcare professionals (eg, care workers, data entry personnel and health information system managers) were considered essential workers. Thus, arrangements were provided to enable them to be present at work, minimising disruptions to routine data collection and entry.^12^

### Outcomes

We sought to include programme indicators related to HIV, malaria and TB services. As HIV indicators are often closely aligned with UNAIDS targets (95-95-95), we used this approach to select malaria and TB indicators for inclusion in our analysis, in order to assess the screening, diagnosis and treatment dimensions, within the limits of available data. These indicators have been validated by a multidisciplinary team (epidemiologists; HIV, malaria and TB programme stakeholders, etc).

A total of nine indicators (three per condition) were included in the final analyses. Thus, for HIV infection, the indicators retained were the (1) number of adults tested for HIV, (2) number of infants born to HIV-positive mothers who underwent PCR testing at 6 weeks for the screening dimension and (3) number of initiations of antiretroviral treatment for the treatment dimension. For malaria control services, the indicators were the (1) number of malaria cases confirmed by rapid diagnostic test (RDT) or thick smear (TS) (diagnostic dimension), (2) number of patients treated with artemether and lumefantrine and (3) number of patients hospitalised for malaria (treatment dimension). Finally, the indicators selected for TB are (1) the number of positive microscopies, (2) the number of positive Xpert tests for *Mycobacterium tuberculosis* (MTB) and (3) the number of TB case notifications. The first two counted for the diagnostic dimension and the last for the treatment dimension.

The detailed definitions of each indicator are given in online supplemental table 1.

10.1136/bmjgh-2023-013679.supp2 Supplementary data

We analysed the absolute number of visits or services provided rather than indicators of service coverage (ie, the proportion of the target population that benefited from a specific service). The latter indicators may be unreliable because they depend on the estimated size of the target population (eg, the population served) as the denominator.^17^

### Statistical analysis

We conducted segmented regression analyses of the interrupted time series by fitting a Poisson regression model with Newey-West SEs to account for autocorrelation and heteroscedasticity.^18 19^ The regression model for the interrupted time series used the following equation:

(eqation 1) $$log\left(E\left(Y\right)\right)=\beta_{0}+\beta_{1}T+\beta_{2}L+\beta_{3}W)$$

*Y* represents the variable (indicator) of interest. *T is* the number of time points since the beginning of the observation period (up to 20 for TB indicators and 36 for HIV and malaria indicators), *L* is an indicator variable worth 1 for time points following the declaration of the COVID-19 pandemic (from April 2020), and *W* represents the number of time points since the declaration of the pandemic (0 for the prepandemic period).

*β0* represents the baseline level of the variable of interest. *β1* represents the average change in *log(E(Y*)) per period before the COVID-19 pandemic. *β2* represents the average change in the level of *log(E(Y*)) immediately after the occurrence of the COVID-19 pandemic (ie, the change in between the last measurement before and the first measurement the month or quarter after the interruption). *β3* represents the average difference in the slope during the COVID-19 pandemic period compared with the pre-COVID-19 pandemic period. *β2* and *β3*, our main parameters of interest, are presented and interpreted as incidence risk ratios (IRRs) with their respective 95% CIs. If the slope change (before and during the COVID-19 pandemic) was not significant (p>0.05), the trend of the indicator remained the same despite the occurrence of the pandemic. A significant increase in the slope during the pandemic (IRR greater than 1 and p<0.05) indicated an upward (positive) trend in the data, while a significant decrease in the slope (IRR less than 1 and p<0.05) indicated a downward (negative) trend.

We used the model to estimate the immediate impact of the occurrence of the COVID-19 pandemic and its effect on the trend. Thus, the main parameters of interest were (1) the change in the level of health services after the declaration of the pandemic and (2) the comparison between prepandemic and pandemic trends.

To account for seasonal changes (eg, rainfall for malaria and vacation periods when visits are generally lower for HIV), we performed a sensitivity analysis (online supplemental files) with two pairs of sine and cosine terms (Fourier terms) included in the model (equation 2).

10.1136/bmjgh-2023-013679.supp1 Supplementary data

(equation 2) $$log\left(E\left(Y\right)\right)=\beta_{0}+\beta_{1}T+\beta_{2}L+\beta_{3}W+\beta_{4}\sin\left(\frac{2\pi t}{12}\right)+\beta_{5}\cos\left(\frac{2\pi t}{12}\right)+\beta_{6}\sin\left(\frac{4\pi t}{12}\right)+\beta_{7}\cos\left(\frac{4\pi t}{12}\right)+\beta_{8}\sin\left(\frac{6\pi t}{12}\right)+\beta_{9}\cos\left(\frac{6\pi t}{12}\right)$$

The data were analysed by using R V.4.2.2 software.

### Patient and public involvement

This study was conducted without patient participation. Patients were not asked to comment on the study protocol and were not consulted to develop patient-relevant results or interpret the results. Patients did not contribute to the writing or editing of this manuscript.

## Results

We analysed a total of nine indicators covering the screening, diagnosis and treatment of HIV, malaria and TB. The variation in service delivery indicators was not homogeneous over the COVID-19 period (table 1). For example, before the pandemic, 196 children born to HIV-positive women were tested by PCR at 6 weeks of age each month, compared with just 172 per month during the COVID-19 period. In contrast, an average of 207 positive cases of TB were diagnosed by the MTB Xpert test per month before the COVID-19 pandemic, whereas during the pandemic, 469 positive cases were diagnosed (table 1).

**Table 1:** HIV, malaria and TB indicators before and during COVID-19 pandemic, Togo, January 2019–December 2020

Characteristic	Observation period		
	Before COVID-19	During COVID-19	Overall
HIV indicators (months)*	**N=15**	**N=21**	**N=36**
Number of ART initiation			
Mean (SD)	986 (344)	1135 (288)	1073 (316)
Median (IQR)	848 (698–1347)	1069 (965–1194)	1045 (834–1272)
Range	589–1596	767–1893	589–1893
Number of persons testing for HIV			
Mean (SD)	38 082 (3645)	42 952 (4693)	40 923 (4881)
Median (IQR)	38 921 (35 292–40 486)	43 004 (40 859–45 874)	41 107 (38 326–44 098)
Range	31 554–43 598	33 320–54 616	31 554–54 616
Number of infants testing for HIV (PCR 1)			
Mean (SD)	196 (30)	172 (41)	182 (38)
Median (IQR)	203 (170–216)	165 (141–210)	184 (150–214)
Range	151–238	108–253	108–253
Malaria indicators (months)^*^	**N=15**	**N=21**	**N=36**
Number of malaria cases confirmed by RDT/TS			
Mean (SD)	126 227 (44 834)	109 299 (27 864)	116 353 (36 322)
Median (IQR)	119 274 (89 500–166 570)	106 112 (85 734–136 540)	111 740 (85 139–141 578)
Range	71 016–188 191	61 403–161 238	61 403–188 191
Number of patients treated with AL			
Mean (SD)	114 554 (39 775)	99 078 (23 643)	105 526 (31 814)
Median (IQR)	110 610 (81 728–149 870)	95 910 (88 734–118 979)	98 360 (84 458–127 102)
Range	63 424–170 134	49 829–133 666	49 829–170 134
Number of in-patient malaria cases			
Mean (SD)	3008 (1214)	3226 (994)	3135 (1080)
Median (IQR)	2857 (2094–3948)	3066 (2442–4174)	3048 (2184–4010)
Range	1336–4998	1380–4698	1336–4998
TB indicators (Quarters)^†^	**N=9**	**N=11**	**N=20**
Number of positive samples examined by microscopy			
Mean (SD)	499 (76)	431 (140)	461 (118)
Median (IQR)	489 (464–508)	391 (322–546)	473 (375–535)
Range	382–645	269–656	269–656
Number of MTB-positive Xpert			
Mean (SD)	207 (158)	469 (144)	351 (198)
Median (IQR)	207 (128–291)	453 (340–578)	340 (244–480)
Range	0–463	270–677	0–677
TB cases notification			
Mean (SD)	653 (47)	663 (112)	659 (87)
Median (IQR)	648 (623–680)	641 (580–716)	644 (606–706)
Range	578–721	521–869	521–869

*Time period: January 2019–December 2021.

†Time period: first quarter 2018 to fourth quarter 2022.

AL, arthemeter lumefantrine; ART, antiretroviral treatment; MTB, *Mycobacterium tuberculosis*; RDT, rapid diagnostic test; TB, Tuberculosis; TS, thick smear.

### Immediate effects after the declaration of the pandemic

Table 2 shows the changes in indicator levels at the time points (month or quarter) just after the onset of the pandemic. Overall, there was a decrease in six of the nine indicators, ranging from 19.3% (IRR 0.807, 95% CI 0.682 to 0.955, p=0.024) in the hospitalisation of patients for malaria to 36.9% (IRR 0.631, 95% CI 0.457 to 0.871, p=0.013) for the diagnosis of TB by MTB Xpert immediately after the declaration of the pandemic.

**Table 2:** Impact of COVID-19 pandemic on HIV, malaria and TB indicators in Togo, Poisson segmented regression models

	Level change at COVID-19 declaration			Trend change during COVID-19 pandemic		
	IRR*	**95% CI**	**P value**	**IRR**	**95%** **CI**	**P value**
HIV indicators†						
PCR tests in infants	0.735	0.628 to 0.861	0.002	1.030	1.015 to 1.046	0.001
Adults tested for HIV	1.062	0.965 to 1.176	0.235	1.013	1.007 to 1.019	<0.001
ART initiation	0.917	0.735 to 1.145	0.456	0.909	0.892 to 0.926	<0.001
Malaria indicators†						
Malaria confirmed cases (RDT/TS)	0.677	0.570 to 0.803	<0.001	0.991	0.978 to 1.004	0.177
Patients treated with AL	0.659	0.567 to 0.765	<0.001	0.998	0.984 to 1.012	0.796
In-patient malaria cases	0.807	0.682 to 0.955	0.024	0.992	0.979 to 1.005	0.228
TB indicators‡						
Positive microscopy	1.097	0.826 to 1.456	0.530	0.919	0.880 to 0.960	0.002
Positive Xpert MTB tests	0.631	0.457 to 0.871	0.013	0.852	0.727 to 0.999	0.065
TB cases notification	0.726	0.657 to 0.802	<0.001	1.035	1.021 to 1.049	0.001

*IRR for the average effect of COVID-19

†Time period: January 2019–December 2021; Autocorrelation addressed using Newey–West SEs to calculate CIs, with lag up to 2.

‡Time period: first quarter 2018 to fourth quarter 2022; Autocorrelation addressed using Newey–West SEs to calculate CIs, with lag up to 3.

AL, arthemeter lumefantrine; ART, antiretroviral treatment; IRR, incidence rate ratio; MTB, *Mycobacterium tuberculosis*; RDT, rapid diagnostic test; TB, Tuberculosis; TS, thick smear.

For HIV, the number of adults tested and the number who initiated antiretroviral therapy (ART) remained constant, while the number of infants who received a PCR test at 6 weeks was significantly reduced by 26.5% (IRR 0.735, 95% CI 0.628 to 0.861, p=0.002).

Immediately after the start of the pandemic, the number of malaria cases confirmed by RDT/TS decreased by 32.3% (IRR 0.677, 95% CI 0.570 to 0.803, p<0.001), and the number of patients hospitalised for malaria management decreased by 19.3% (IRR 0.807, 95% CI 0.682 to 0.955, p=0.024). Finally, the number of patients treated for uncomplicated malaria with arthemeter lumefantrine (artemisinin-based combination therapy) also decreased by 34.1% (IRR 0.659, 95% CI 0.567 to 0.765, p<0.001) (table 2).

For two of the three TB indicators, the levels decreased immediately after the start of the pandemic. The number of TB case notifications decreased by 27.4% (IRR 0.726, 95% CI 0.657 to 0.802, p<0.001), and the number of Xpert diagnoses decreased by 36.9% (IRR 0.631, 95% CI 0.457 to 0.871, p=0.013) (table 2).

### Pandemic trend

**Figure 1:**
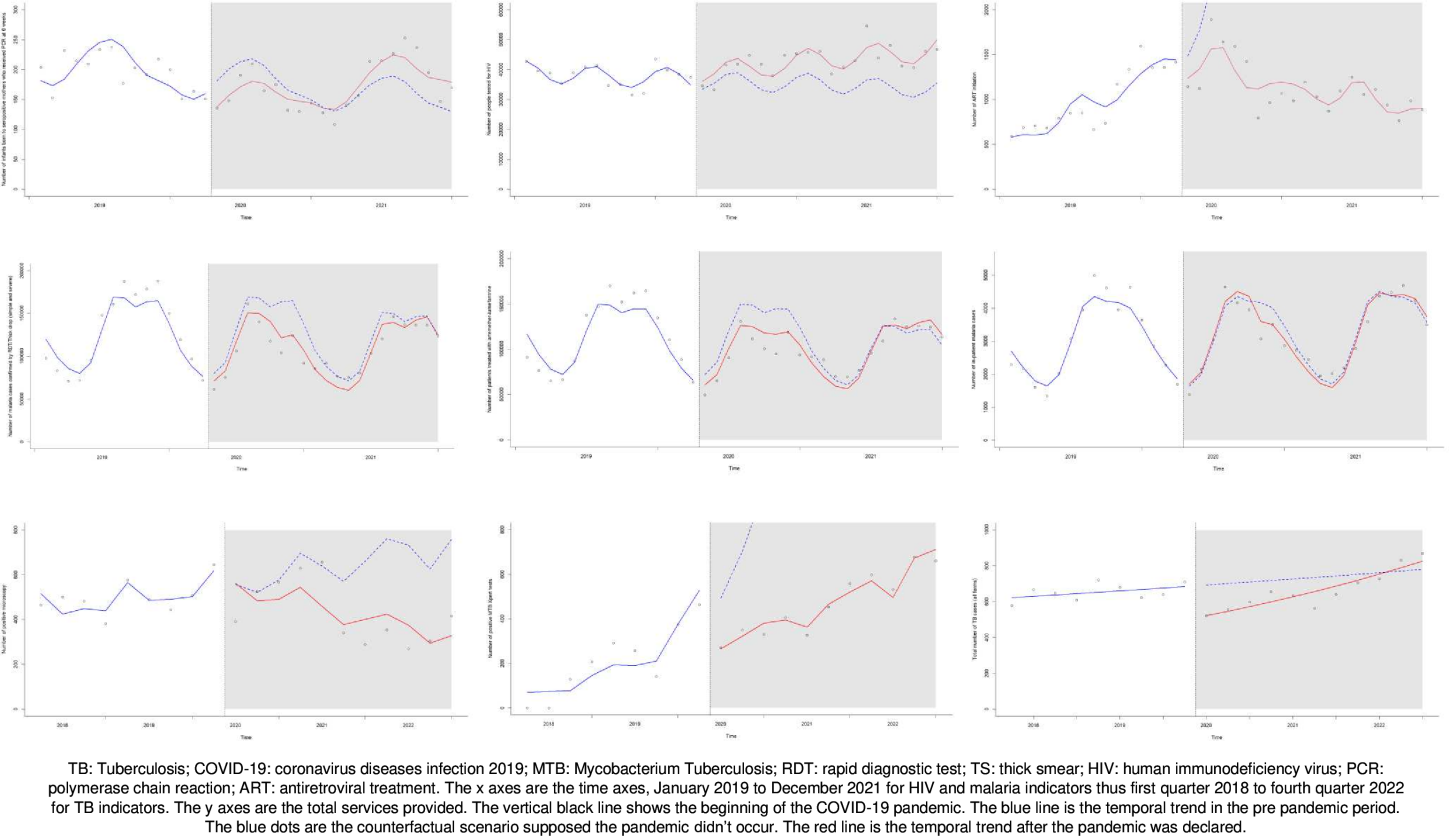
The observed and predicted trends over the study period for all nine indicators analysed.

A comparison of the observed and predicted trends by the segmented Poisson regression model showed that the trend appeared to have remained constant between the prepandemic and pandemic periods for all the malaria indicators (table 2 and figure 1).

A significant downward monthly trend was observed in ART initiation (IRR 0.909, 95% CI 0.892 to 0.926, p<0.001) and positive TB microscopy (IRR 0.919, 95% CI 0.880 to 0.960, p=0.002). Elsewhere, an upward monthly trend was observed in HIV PCR testing of infants at 6 weeks (IRR 1.030, 95% CI 1.015 to 1.046, p=0.001), HIV tests among adults (IRR 1.013, 95% CI 1.007 to 1.019, p<0.001) and TB case notifications (IRR 1.035, 95% CI 1.021 to 1.049, p=0.001).

## Discussion

### Overall effect of the COVID-19 pandemic

This study described the effect of the COVID-19 pandemic on nine indicators of the diagnosis and treatment of HIV infection, malaria and TB in Togo. We found a significant decrease in the levels of all malaria indicators immediately after the onset of COVID-19, with a constant trend observed during the pandemic. The immediate decrease in HIV PCR testing among infants at 6 weeks and in the number of TB case notifications was followed by a gradual improvement in the following months during the pandemic period. These findings suggest that HIV, malaria and TB services were generally maintained over time.

While the COVID-19 pandemic received much attention in early 2020, threats posed by other diseases persisted in most countries. In a survey of 105 countries in 2020, the WHO reported that epidemic detection and control of these diseases suffered from a focus on COVID-19 in 45% of countries.^20^ The Global Fund to fight HIV, malaria and TB estimated that TB and HIV testing declined by 18%–22% in countries supported by the Fund.^21^ Given the weakness of Africa’s healthcare systems, the challenges posed by endemic diseases such as the big three and other constraints (eg, inadequate healthcare funding and shortage of human resources), initial forecasts suggested that the onset of the COVID-19 pandemic would cause an epidemiological and humanitarian catastrophe on the continent.^22^ However, the evolution of the COVID-19 pandemic and its mixed effects on the continent ultimately puzzled many public health experts.^22^

Using data from the Ministry of Health in Togo, we described disruptions in six out of nine indicators immediately after the onset of the pandemic. On the other hand, there was an upward trend in three indicators during the pandemic period, while four indicators remained stable over time. These results provide information about the resilience of HIV, malaria and TB control programmes in Togo. There are various possible explanations for these results. First, these programmes have been implemented for decades, and they have developed a harmonious operational structure that enabled them to adapt to the crisis and challenges of COVID-19. Second, multiform financial and structural support was provided by external partners, mainly the Global Fund, to mitigate the effects of the pandemic on the fight against these three diseases in the months following the COVID-19 outbreak. Indeed, the Global Fund’s COVID-19 response mechanism enabled 18% of beneficiary countries, including Togo, to implement strategies to mitigate the impact of COVID-19 on HIV, malaria and TB programmes.^23^ Third, the extent of the effect can be correlated with the scale of the local epidemic and the intensity of response measures.^10^ Togo is a relatively small country (by area and population) where the COVID-19 epidemic was of relatively low magnitude (39 506 cases and 290 deaths as of 7 July 2023). In addition, the response measures were fairly vigorous and prompt, earning Togo a place at the top of the continental table for its management of the pandemic in 2021.^10^

### Effects on HIV services

In line with UNAIDS targets for the eradication of HIV/AIDS, HIV testing is the first step in initiating the continuum of HIV care.^24^ In fact, a decrease in screening alters the whole care cascade and would contribute to slowing efforts in the fight against HIV. Numerous studies have reported disruptions in HIV testing during the COVID-19 pandemic of up to 50%.^9 10 20^ In our study, we observed that diagnoses of HIV infection in adults in Togo were maintained over time. In 2019, the WHO issued recommendations on integrating innovative HIV testing approaches into national strategies.^25^ In accordance with these recommendations, various projects initiated with PEPFAR support have progressively deployed certain approaches, such as self-testing, index testing, social network-based HIV testing approaches and community-based screening, starting with specific key populations.^26^ These new testing approaches alone have contributed to more than 48% of the new cases detected in 2022 at PEPFAR sites.^27^ The implementation of these screening approaches could, therefore, have contributed to our findings and improved the indicators in Togo. In view of their role in reaching screening targets, Togo’s National HIV and AIDS Strategic Plan 2021–2025 calls for these approaches to be scaled up. These strategies could be considered in the fight against HIV in similar contexts.

Elsewhere, a significant decrease in HIV PCR tests in infants was noted, followed by a significant upward trend during the pandemic period compared with the prepandemic period. In Togo, the onset of the COVID-19 pandemic gave rise to two major problems, namely, access to children’s PCR samples and the availability of PCR materials.^28^ Samples from infants for HIV PCR testing were taken exclusively in health facilities. However, as in many countries, the government of Togo implemented physical distancing measures beginning 19 March 2020, by placing nationwide bans on all public gatherings.^12^ Subsequently, cordoning off of certain towns and public transport restrictions were implemented.^12^ All of these restrictions and the fear of being infected by COVID-19 limited the access of mothers and children to these health facilities, which may have postponed their PCR test appointments, particularly during the early period of the pandemic.^28^ In the future, this problem could be partly solved by extending the community-based HIV screening strategy currently being rolled out for adults to infants.

In our study, although there was no immediate decline in ART initiation at the onset of the pandemic, this indicator showed a downward trend during the pandemic period. Different scenarios are described in the literature. Indeed, Dorward *et al,* in an ITSA, reported an almost 50% decrease in ART initiation in KwaZulu-Natal, South Africa, at the start of the COVID-19 lockdown followed by gradual improvement towards prelockdown levels.^10^ With similar findings in rural South Africa, other authors have suggested that the increase in HIV service utilisation might be due to people rushing to obtain ART in anticipation of potential future mobility restrictions or drug shortages.^29^ Using the same methodological approach, Osei *et al* in Ghana showed that the number of HIV-positive patients starting ART increased by approximately 39% in the first month of the pandemic but decreased by an average of 14% per month in the following three months of 2020.^30^ The authors argued that the declines in ART initiation in some months of the pandemic could be associated with occasional shortages of antiretroviral drugs.^30^ Although the epidemiological contexts of HIV in Ghana and Togo are similar, an ART shortage was not reported in Togo at the start of the pandemic.^28 31^

### Effect on malaria services

We described a disruption in malaria diagnosis, hospitalisation and treatment in April 2020 following the onset of the COVID-19 pandemic, during which the trend remained constant during the pandemic compared with that of the prepandemic period. Similar disruptions have been described in other contexts^20 32^ and in neighbouring countries such as Ghana.^9^ Since fever is a common symptom of both diseases, one of the reasons for the decrease in healthcare facility attendance was the fear of being misdiagnosed^33^ and being quarantined or isolated. Furthermore, patients may have opted for self-medication and avoided the healthcare system; there is evidence of a high level of self-medication during the COVID-19 period in Togo.^34^ The similarity of symptoms is an important factor that gave rise to fear among healthcare providers in charge of malaria patients.^20 32^ Thus, the provision of malaria-related services may have declined in the early days of COVID-19. According to Arsenault *et al*, such short-term disruptions combined with probable interruptions in prevention activities (such as mosquito nets and insecticide spraying) could still have led to an increase in the number of malaria deaths, which is challenging for malaria-endemic countries.^9^ It would be useful to consider effective communication plans during pandemics to provide the population with the right information to support care-seeking behaviour.

### Effect on TB services

TB services worldwide were affected by the onset of the COVID-19 pandemic. Almost half (42%) of the countries surveyed in the WHO PULSE 2020 reported a partial interruption of TB case detection and treatment services.^20^ In Malawi, an analysis similar to ours reported a substantial immediate decline in TB case notifications concurrent with the start of the COVID-19 pandemic.^35^ We made concordant observations. In Togo, the majority of biological TB diagnoses are made by microscopy despite the progressive deployment of the Xpert MTB platform. The number of diagnoses made by microscopy significantly decreased in the months following the onset of the pandemic. Additionally, the number of diagnoses by Xpert decreased significantly immediately after the start of the pandemic. One hypothesis is that at the beginning of the pandemic, these platforms were mobilised for COVID-19 screening. Fewer diagnoses meant less treatment; we identified a 17% decrease in TB case notifications at the start of the pandemic. The decreases reported in this and other studies may suggest the influence of many other factors, such as real reductions in transmission associated with physical distancing and the use of face masks^30 36^ or the reallocation and reprioritisation of healthcare staff, funding and medical supplies from TB control programmes to COVID-19 control, which could have contributed to fewer field activities.^30^

### Perspectives for future research

Further research is needed to understand the full indirect effects of the pandemic on healthcare provision. For example, our analysis did not take into account changes in the quality of healthcare, which was probably negatively affected during the pandemic. Similarly, it would be relevant to explore other health services, such as sexual and reproductive health services,^37^ immunisation activities and care for non-communicable diseases, to obtain a broader picture of the effect of the pandemic on Togo’s health system. Finally, it is important to clarify the factors responsible for disruptions to healthcare services to better prepare for future pandemics.^9^

### Strengths and limitations

To the best of our knowledge, this is the first study to assess the effect of the COVID-19 pandemic on healthcare utilisation within the Togo healthcare system. The main strength of this study is that we used nationally representative healthcare system data, allowing us to generalise the results to the entire target population.

The findings of this study must, however, be interpreted in light of its limitations. First, despite the relevance and increasing use of the ITSA design,^38 39^ there are numerous ways to analyse ITSA studies and report results across a range of healthcare studies.^39 40^ Furthermore, we used a single ITSA. In a systematic review by Ayouni *et al*, most studies evaluating the impact of COVID-19 did not include control groups.^41^ Indeed, in some cases, the non-existence of a suitable control population (not affected by the intervention) is noted in the literature.^42^ In such cases, ITSA may still be performed for the intervention group.^42^ Second, it is possible that health measures/policies implemented between April 2020 and December 2021 could have constituted additional interruptions in the normal temporal pattern. Future studies should include other interruption points to refine model estimates. Third, we included a relatively limited number of indicators, and TB indicators were described on a quarterly basis, which may have affected the estimation by reducing the number of estimation points. Despite these limitations, this study provides relevant information on how the COVID-19 pandemic affected HIV, malaria and TB indicators in Togo, which is useful for programme planning and preparing for future crises.

## Conclusion

HIV, malaria and TB services were generally maintained over time in Togo despite the COVID-19 pandemic. However, given the decline in some indicators immediately after the declaration of the pandemic, there is room for improvement in the preparedness of the healthcare system to cope with future epidemics.

## Data Availability

Data may be obtained from a third party and are not publicly available.
