# Supplementary material for: Effect of the COVID-19 pandemic on HIV, malaria and tuberculosis indicators in Togo: an interrupted time series analysis
Source: BMJ Glob Health. 2024 Apr 3;9(4):e013679. doi: 10.1136/bmjgh-2023-013679 (PMC11002417; doi:10.1136/bmjgh-2023-013679)
Supplement: Supplementary data [file bmjgh-2023-013679supp002.pdf]

Supplementary Table

	Indicators	Explications
**HIV**		
	Infants testing for HIV (PCR)	Number of infants born to HIV infected mothers who underwent PCR test at 6 weeks of age (with results)
	Person testing for HIV	Number of people tested for HIV
	PLWHIV initiating ART	Number of HIV infected people initiating ART
**Tuberculosis (TB)**		
	Positive samples examined by microscopy	Number positive samples examined by microscopy for the diagnosis of drug-susceptible tuberculosis
	MTB-positive Xpert	Number of MTB-positive Xpert tests in new cases
	TB cases	Number of people diagnosed with TB and initiating the treatment (no matter the clinical form of TB)
**Malaria**		
	Malaria in-patients	Number of inpatient malaria cases
	Patients treated with AL	Number of patients treated with artemether-lumefantrine
	Malaria cases confirmed by RDT/TS	Number of malaria cases confirmed by RDT/TS (simple and severe)

TB: Tuberculosis; COVID-19: coronavirus diseases infection 2019; MTB: Mycobacterium Tuberculosis; RDT: rapid diagnostic test; TS: thick smear; HIV: human immunodeficiency virus; PCR: polymerase chain reaction; ART: antiretroviral treatment; AL: arthemeter-lumefantrine
