# Supplementary material for: Effect of the COVID-19 pandemic on HIV, malaria and tuberculosis indicators in Togo: an interrupted time series analysis
Source: BMJ Glob Health. 2024 Apr 3;9(4):e013679. doi: 10.1136/bmjgh-2023-013679 (PMC11002417; doi:10.1136/bmjgh-2023-013679)
Supplement: Supplementary data [file bmjgh-2023-013679supp001.pdf]

BIG_3_Model_assumptions_Sensitivity_Goodness_of_Fit

2023-10-18

```
##
## Attachement du package : 'dplyr'

## Les objets suivants sont masqués depuis 'package:stats':
##
##     filter, lag

## Les objets suivants sont masqués depuis 'package:base':
##
##     intersect, setdiff, setequal, union

## -- Attaching core tidyverse packages ------------------------ tidyverse 2.0.0 --
## v forcats   1.0.0     v readr     2.1.4
## v ggplot2   3.4.4     v stringr   1.5.0
## v lubridate 1.9.3     v tibble    3.2.1
## v purrr     1.0.2     v tidyr     1.3.0
## -- Conflicts ------------------------------------------ tidyverse_conflicts() --
## x dplyr::filter() masks stats::filter()
## x dplyr::lag()    masks stats::lag()
## i Use the conflicted package (<http://conflicted.r-lib.org/>) to force all conflicts to become errors
## Le chargement a nécessité le package : survival
##
## Package epiR 2.0.65 is loaded
##
## Type help(epi.about) for summary information
##
## Type browseVignettes(package = 'epiR') to learn how to use epiR for applied epidemiological analyses
##
##
##
##
## Registered S3 method overwritten by 'quantmod':
##   method            from
##   as.zoo.data.frame zoo
##
##
## Attachement du package : 'forecast'
##
##
## L'objet suivant est masqué depuis 'package:ggpubr':
##
##     gghistogram
##
##
```


Konu YR, *et al. BMJ Glob Health* 2024; 9:e013679. doi: 10.1136/bmjgh-2023-013679

```
## Time Series Modeling for Air Pollution and Health (0.6-1)
##
## Le chargement a nécessité le package : zoo
##
##
## Attachement du package : 'zoo'
##
##
## Les objets suivants sont masqués depuis 'package:base':
##
##     as.Date, as.Date.numeric
```

TB indicators

Number Positive samples examined by microscopy for the diagnosis of drug-susceptible tuberculosis

```
### E(Y)=Var(Y)=Lamda assumption verification
mean(tb$micro)
```

```
## [1] 461.4
```

```
var(tb$micro)
```

```
## [1] 13916.36
```

```
### Poisson segmented regression
poi <- glm(micro ~ time + level + trend, data=tb, family = "poisson")
pois <- glm(micro ~ time + level + trend + harmonic(time, 4, 4), data=tb, family = "poisson")
summary(poi)
```

```
##
## Call:
## glm(formula = micro ~ time + level + trend, family = "poisson",
##     data = tb)
##
## Coefficients:
##              Estimate Std. Error z value Pr(>|z|)
## (Intercept)  6.080226   0.033454 181.747  < 2e-16 ***
## time         0.025982   0.005788   4.489 7.16e-06 ***
## level        0.064224   0.039553   1.624    0.104
## trend       -0.080595   0.007416 -10.868  < 2e-16 ***
## ---
## Signif. codes:  0 '***' 0.001 '**' 0.01 '*' 0.05 '.' 0.1 ' ' 1
##
## (Dispersion parameter for poisson family taken to be 1)
##
##     Null deviance: 583.41  on 19  degrees of freedom
## Residual deviance: 373.55  on 16  degrees of freedom
```

```
## AIC: 540.34
##
## Number of Fisher Scoring iterations: 4
```

```r
summary(pois)
```

```
##
## Call:
## glm(formula = micro ~ time + level + trend + harmonic(time, 4,
##     4), family = "poisson", data = tb)
##
## Coefficients: (3 not defined because of singularities)
##                         Estimate Std. Error z value Pr(>|z|)
## (Intercept)            6.047e+00  3.392e-02 178.289  < 2e-16 ***
## time                   2.350e-02  5.942e-03   3.955 7.65e-05 ***
## level                  9.269e-02  4.080e-02   2.272  0.02311 *
## trend                 -8.468e-02  7.477e-03 -11.325  < 2e-16 ***
## harmonic(time, 4, 4)1  9.317e-02  2.028e-02   4.595 4.33e-06 ***
## harmonic(time, 4, 4)2 -4.766e+13  1.612e+13  -2.957  0.00311 **
## harmonic(time, 4, 4)3         NA         NA      NA       NA
## harmonic(time, 4, 4)4 -1.987e+13  8.833e+12  -2.249  0.02450 *
## harmonic(time, 4, 4)5 -1.716e-02  1.553e-02  -1.105  0.26920
## harmonic(time, 4, 4)6 -8.391e-02  2.256e-02  -3.720  0.00020 ***
## harmonic(time, 4, 4)7         NA         NA      NA       NA
## harmonic(time, 4, 4)8         NA         NA      NA       NA
## ---
## Signif. codes:  0 '***' 0.001 '**' 0.01 '*' 0.05 '.' 0.1 ' ' 1
##
## (Dispersion parameter for poisson family taken to be 1)
##
##     Null deviance: 583.41  on 19  degrees of freedom
## Residual deviance: 300.39  on 11  degrees of freedom
## AIC: 477.18
##
## Number of Fisher Scoring iterations: 4
```

```r
anova(poi, pois, test = "Chisq")
```

```
## Analysis of Deviance Table
##
## Model 1: micro ~ time + level + trend
## Model 2: micro ~ time + level + trend + harmonic(time, 4, 4)
##   Resid. Df Resid. Dev Df Deviance  Pr(>Chi)
## 1        16     373.55
## 2        11     300.39  5    73.16 2.251e-14 ***
## ---
## Signif. codes:  0 '***' 0.001 '**' 0.01 '*' 0.05 '.' 0.1 ' ' 1
```

```r
### Checking for autocorrelation and seasonality in the residuals
res_poi <- residuals(pois, type="deviance")
ggtsdisplay(res_poi)
```


Konu YR, et al. BMJ Glob Health 2024; 9:e013679. doi: 10.1136/bmjgh-2023-013679

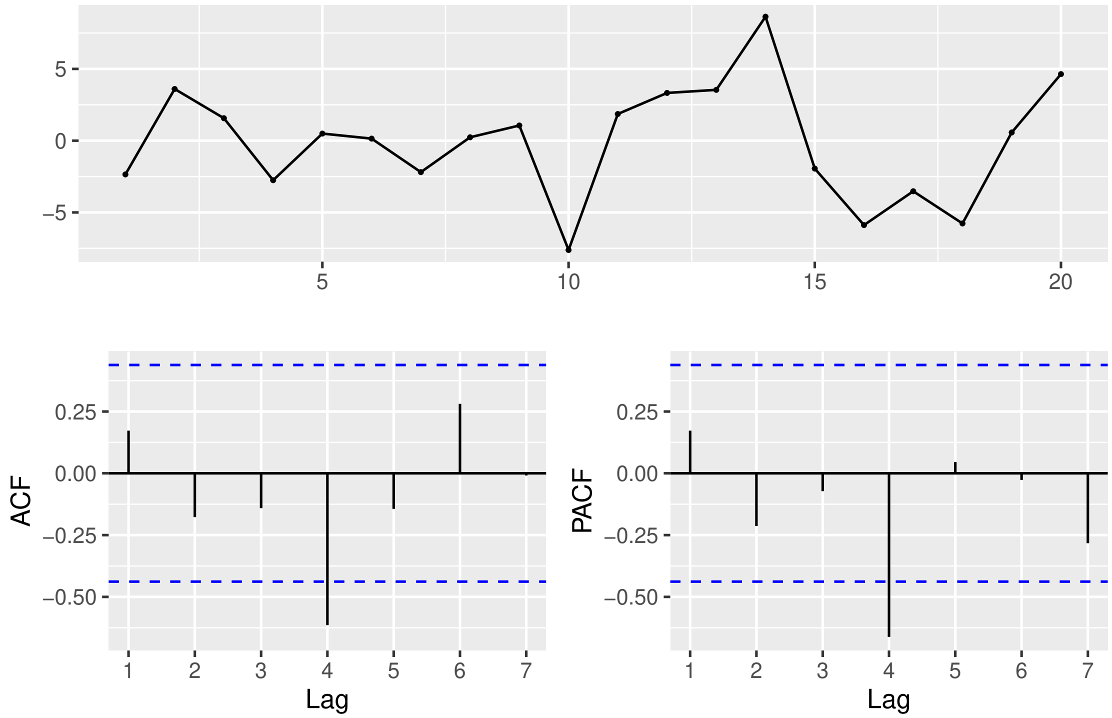


```
!is.null(tbats(ts(res_poi, frequency=4))$seasonal) #No Seasonality in the residuals
```

```
## [1] FALSE
```

```
### Goodness of fit
with(pois, cbind(res.deviance = deviance, df = df.residual, rapport = deviance/df.residual))
```

```
##      res.deviance df rapport
## [1,]     300.3858 11 27.3078
```

```
with(pois, cbind(res.deviance = deviance, df = df.residual, p = pchisq(deviance, df.residual, lower.tail=FALSE)
```

```
##      res.deviance df            p
## [1,]     300.3858 11 7.265009e-58
```

Number Positive MTB Xpert tests

```
### E(Y)=Var(Y)=Lamda assumption verification
mean(tb$xpert)
```

```
## [1] 351.2
```


Konu YR, *et al. BMJ Glob Health* 2024; 9:e013679. doi: 10.1136/bmjgh-2023-013679

```r
var(tb$xpert)
```

```
## [1] 39312.8
```

```r
### Poisson segmented regression
#### Poisson Model
poi <- glm(xpert ~ time + level + trend, data=tb, family = "poisson")
pois <- glm(xpert ~ time + level + trend + harmonic(time, 2, 4), data=tb, family = "poisson")
summary(poi)
```

```
##
## Call:
## glm(formula = xpert ~ time + level + trend, family = "poisson",
##     data = tb)
##
## Coefficients:
##             Estimate Std. Error z value Pr(>|z|)
## (Intercept)  3.75421    0.07218   52.01   <2e-16 ***
## time         0.26941    0.01030   26.16   <2e-16 ***
## level       -0.59639    0.04775  -12.49   <2e-16 ***
## trend       -0.18107    0.01124  -16.11   <2e-16 ***
## ---
## Signif. codes:  0 '***' 0.001 '**' 0.01 '*' 0.05 '.' 0.1 ' ' 1
##
## (Dispersion parameter for poisson family taken to be 1)
##
##     Null deviance: 2737.49  on 19  degrees of freedom
## Residual deviance:  537.55  on 16  degrees of freedom
## AIC: 684.24
##
## Number of Fisher Scoring iterations: 5
```

```r
summary(pois)
```

```
##
## Call:
## glm(formula = xpert ~ time + level + trend + harmonic(time, 2,
##     4), family = "poisson", data = tb)
##
## Coefficients:
##                         Estimate Std. Error z value Pr(>|z|)
## (Intercept)            3.855e+00  7.063e-02  54.579  < 2e-16 ***
## time                   2.454e-01  1.031e-02  23.797  < 2e-16 ***
## level                 -4.604e-01  5.021e-02  -9.170  < 2e-16 ***
## trend                 -1.602e-01  1.126e-02 -14.228  < 2e-16 ***
## harmonic(time, 2, 4)1  2.081e-01  3.332e-02   6.245 4.23e-10 ***
## harmonic(time, 2, 4)2  5.575e+13  1.034e+13   5.392 6.97e-08 ***
## harmonic(time, 2, 4)3  1.024e-01  1.782e-02   5.748 9.01e-09 ***
## harmonic(time, 2, 4)4  6.241e-02  2.261e-02   2.760  0.00578 **
## ---
## Signif. codes:  0 '***' 0.001 '**' 0.01 '*' 0.05 '.' 0.1 ' ' 1
##
```

```
## (Dispersion parameter for poisson family taken to be 1)
##
##     Null deviance: 2737.49  on 19  degrees of freedom
## Residual deviance:  461.89  on 12  degrees of freedom
## AIC: 616.57
##
## Number of Fisher Scoring iterations: 6
```

```
anova(poi, pois, test = "Chisq")
```

```
## Analysis of Deviance Table
##
## Model 1: xpert ~ time + level + trend
## Model 2: xpert ~ time + level + trend + harmonic(time, 2, 4)
##   Resid. Df Resid. Dev Df Deviance  Pr(>Chi)
## 1        16     537.55
## 2        12     461.89  4   75.669 1.439e-15 ***
## ---
## Signif. codes:  0 '***' 0.001 '**' 0.01 '*' 0.05 '.' 0.1 ' ' 1
```

```
### Checking for autocorrelation and seasonality in the residuals
res_poi <- residuals(pois, type="deviance")
ggtsdisplay(res_poi)
```

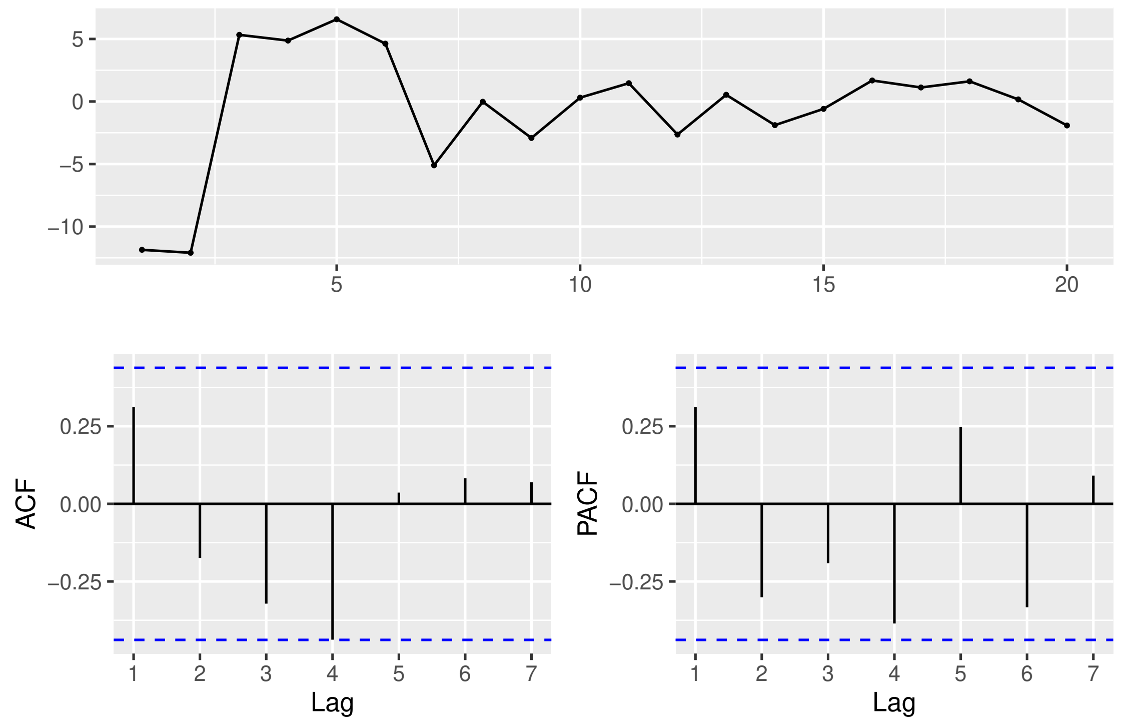

Konu YR, *et al. BMJ Glob Health* 2024; 9:e013679. doi: 10.1136/bmjgh-2023-013679

```
!is.null(tbats(ts(res_poi, frequency=4))$seasonal) #No Seasonality in the residuals
```

```
## [1] FALSE
```

```
### Goodness of fit
with(pois, cbind(res.deviance = deviance, df = df.residual, rapport = deviance/df.residual))
```

```
##      res.deviance df  rapport
## [1,]     461.8854 12 38.49045
```

```
with(pois, cbind(res.deviance = deviance, df = df.residual, p = pchisq(deviance, df.residual, lower.tail=FALSE)
```

```
##      res.deviance df            p
## [1,]     461.8854 12 2.822698e-91
```

Total number of cases, all forms combined

```
### E(Y)=Var(Y)=Lamda assumption verification
mean(tb$cas)
```

```
## [1] 658.55
```

```
var(tb$cas)
```

```
## [1] 7494.997
```

```
### Poisson segmented regression
poi <- glm(cas ~ time + level + trend, data=tb, family = "poisson")
pois <- glm(cas ~ time + level + trend + harmonic(time, 2, 4), data=tb, family = "poisson")
summary(poi)
```

```
##
## Call:
## glm(formula = cas ~ time + level + trend, family = "poisson",
##     data = tb)
##
## Coefficients:
##              Estimate Std. Error z value Pr(>|z|)
## (Intercept)  6.421213   0.028799 222.964  < 2e-16 ***
## time         0.011877   0.005055   2.350   0.0188 *
## level       -0.317403   0.035764  -8.875  < 2e-16 ***
## trend        0.034134   0.006280   5.436 5.46e-08 ***
## ---
## Signif. codes:  0 '***' 0.001 '**' 0.01 '*' 0.05 '.' 0.1 ' ' 1
##
## (Dispersion parameter for poisson family taken to be 1)
##
##     Null deviance: 210.629  on 19  degrees of freedom
```


Konu YR, *et al. BMJ Glob Health* 2024; 9:e013679. doi: 10.1136/bmjgh-2023-013679

```
## Residual deviance:  50.764  on 16  degrees of freedom
## AIC: 225.17
##
## Number of Fisher Scoring iterations: 3
```

```
summary(pois)
```

```
##
## Call:
## glm(formula = cas ~ time + level + trend + harmonic(time, 2,
##     4), family = "poisson", data = tb)
##
## Coefficients:
##                          Estimate Std. Error z value Pr(>|z|)
## (Intercept)             6.414e+00  2.907e-02 220.614  < 2e-16 ***
## time                    1.266e-02  5.115e-03   2.475   0.0133 *
## level                  -3.206e-01  3.667e-02  -8.743  < 2e-16 ***
## trend                   3.432e-02  6.287e-03   5.458 4.81e-08 ***
## harmonic(time, 2, 4)1   2.774e-02  1.726e-02   1.607   0.1079
## harmonic(time, 2, 4)2   7.593e+11  6.167e+12   0.123   0.9020
## harmonic(time, 2, 4)3 -1.390e-02  1.274e-02  -1.091   0.2754
## harmonic(time, 2, 4)4 -9.492e-04  1.275e-02  -0.074   0.9407
## ---
## Signif. codes:  0 '***' 0.001 '**' 0.01 '*' 0.05 '.' 0.1 ' ' 1
##
## (Dispersion parameter for poisson family taken to be 1)
##
##     Null deviance: 210.629  on 19  degrees of freedom
## Residual deviance:  45.049  on 12  degrees of freedom
## AIC: 227.46
##
## Number of Fisher Scoring iterations: 3
```

```
anova(poi, pois, test="Chisq")
```

```
## Analysis of Deviance Table
##
## Model 1: cas ~ time + level + trend
## Model 2: cas ~ time + level + trend + harmonic(time, 2, 4)
##   Resid. Df Resid. Dev Df Deviance Pr(>Chi)
## 1        16     50.764
## 2        12     45.049  4   5.7153   0.2214
```

```
### Checking for autocorrelation and seasonality in the residuals
res_poi <- residuals(poi, type="deviance")
ggtsdisplay(res_poi)
```

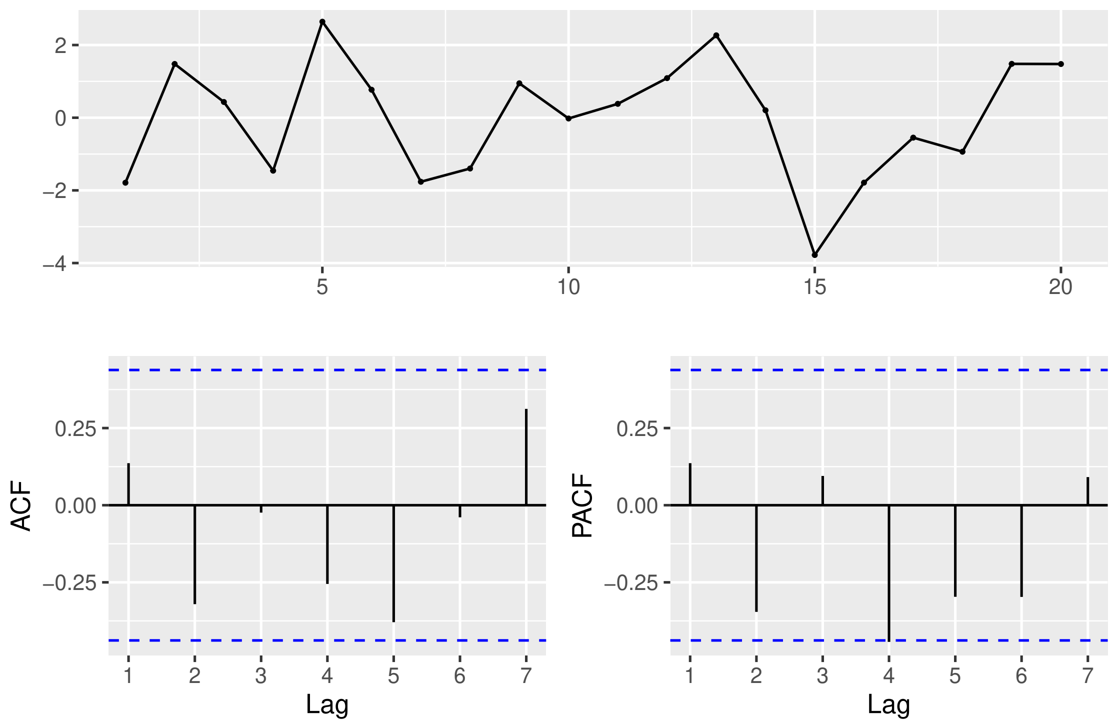


```
!is.null(tbats(ts(res_poi, frequency=4))$seasonal) #No Seasonality in the residuals
```

```
## [1] FALSE
```

```
### Goodness of fit
with(pois, cbind(res.deviance = deviance, df = df.residual, rapport = deviance/df.residual))
```

```
##      res.deviance df  rapport
## [1,]      45.0489 12 3.754075
```

```
with(pois, cbind(res.deviance = deviance, df = df.residual, p = pchisq(deviance, df.residual, lower.tail=FALSE)
```

```
##      res.deviance df           p
## [1,]      45.0489 12 1.01081e-05
```

Malaria Indicators

Number of malaria cases confirmed by RDT/GE Togo

```
### E(Y)=Var(Y)=Lamda assumption verification
mean(palu$t_tdr_ge)
```

```
## [1] 116352.7
```

```r
var(palu$t_tdr_ge)
```

```
## [1] 1319320473
```

```r
### Poisson segmented regression
poi <- glm(t_tdr_ge ~ time + level + trend, data=palu, family = "poisson")
pois <- glm(t_tdr_ge ~ time + level + trend + harmonic(time, 3, 12), data=palu, family = "poisson")
summary(poi) ; summary(pois)
```

```
##
## Call:
## glm(formula = t_tdr_ge ~ time + level + trend, family = "poisson",
##     data = palu)
##
## Coefficients:
##               Estimate Std. Error z value Pr(>|z|)
## (Intercept) 11.5590436  0.0015961 7241.94   <2e-16 ***
## time         0.0227465  0.0001687  134.84   <2e-16 ***
## level       -0.4480598  0.0019413 -230.80   <2e-16 ***
## trend       -0.0094356  0.0002010  -46.95   <2e-16 ***
## ---
## Signif. codes:  0 '***' 0.001 '**' 0.01 '*' 0.05 '.' 0.1 ' ' 1
##
## (Dispersion parameter for poisson family taken to be 1)
##
##     Null deviance: 392499  on 35  degrees of freedom
## Residual deviance: 337988  on 32  degrees of freedom
## AIC: 338480
##
## Number of Fisher Scoring iterations: 4
```

```
##
## Call:
## glm(formula = t_tdr_ge ~ time + level + trend + harmonic(time,
##     3, 12), family = "poisson", data = palu)
##
## Coefficients:
##                          Estimate Std. Error  z value Pr(>|z|)
## (Intercept)            11.6479997  0.0018011 6467.241  < 2e-16 ***
## time                    0.0139931  0.0001976   70.823  < 2e-16 ***
## level                  -0.3905741  0.0021594 -180.872  < 2e-16 ***
## trend                  -0.0092006  0.0002189  -42.031  < 2e-16 ***
## harmonic(time, 3, 12)1 -0.3920376  0.0007574 -517.606  < 2e-16 ***
## harmonic(time, 3, 12)2  0.0569071  0.0007260   78.382  < 2e-16 ***
## harmonic(time, 3, 12)3 -0.0434963  0.0007084  -61.397  < 2e-16 ***
## harmonic(time, 3, 12)4  0.0073524  0.0007497    9.807  < 2e-16 ***
## harmonic(time, 3, 12)5  0.0929198  0.0007151  129.941  < 2e-16 ***
## harmonic(time, 3, 12)6 -0.0031517  0.0007086   -4.447 8.69e-06 ***
## ---
## Signif. codes:  0 '***' 0.001 '**' 0.01 '*' 0.05 '.' 0.1 ' ' 1
```

```
##
## (Dispersion parameter for poisson family taken to be 1)
##
##     Null deviance: 392499  on 35  degrees of freedom
## Residual deviance:  37253  on 26  degrees of freedom
## AIC: 37757
##
## Number of Fisher Scoring iterations: 4
```

```
anova(poi, pois, test = "Chisq")
```

```
## Analysis of Deviance Table
##
## Model 1: t_tdr_ge ~ time + level + trend
## Model 2: t_tdr_ge ~ time + level + trend + harmonic(time, 3, 12)
##   Resid. Df Resid. Dev Df Deviance  Pr(>Chi)
## 1        32     337988
## 2        26      37253  6   300735 < 2.2e-16 ***
## ---
## Signif. codes:  0 '***' 0.001 '**' 0.01 '*' 0.05 '.' 0.1 ' ' 1
```

```
#### Checking for correlation, autocorrelation and seasonality in the residuals
res_poi <- residuals(pois, type="deviance")
ggtsdisplay(res_poi)
```

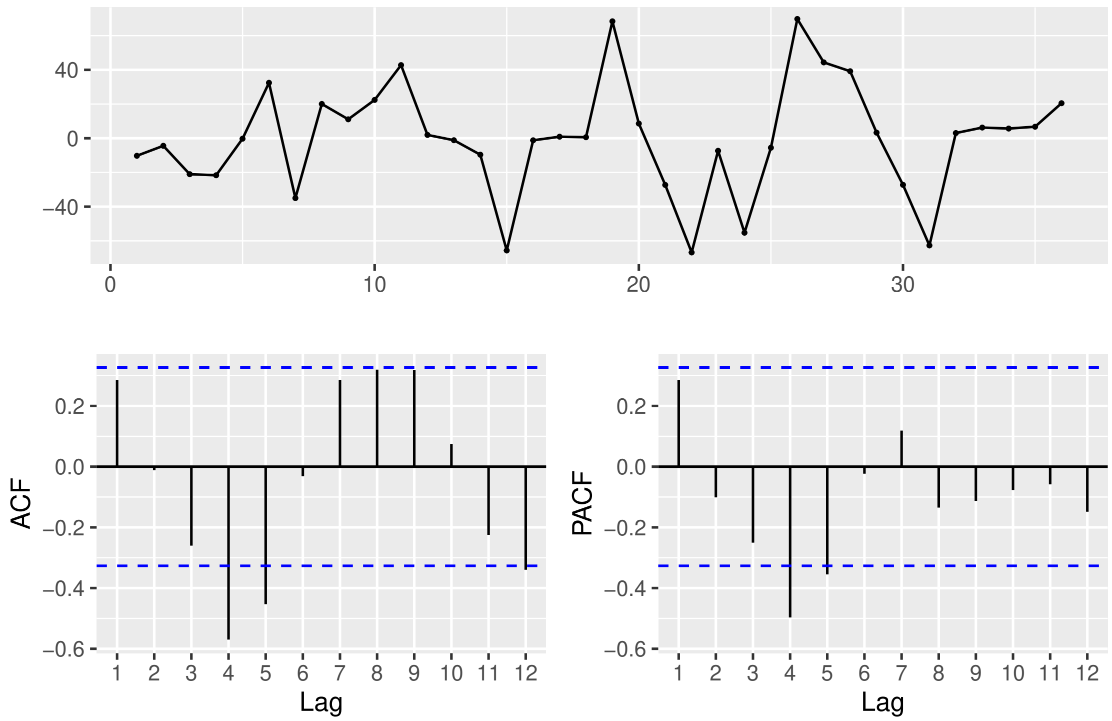

Konu YR, *et al. BMJ Glob Health* 2024; 9:e013679. doi: 10.1136/bmjgh-2023-013679

```
!is.null(tbats(ts(res_poi, frequency=12))$seasonal) #No Seasonality in the residuals
```

```
## [1] FALSE
```

```
### Goodness of fit
with(pois, cbind(res.deviance = deviance, df = df.residual, rapport = deviance/df.residual))
```

```
##      res.deviance df  rapport
## [1,]      37252.7 26 1432.796
```

```
with(pois, cbind(res.deviance = deviance, df = df.residual, p = pchisq(deviance, df.residual, lower.tail=FALSE)
```

```
##      res.deviance df p
## [1,]      37252.7 26 0
```

Number of patients treated with artemether-lumefantrine Togo

```
### E(Y)=Var(Y)=Lamda assumption verification
mean(palu$t_cta)
```

```
## [1] 105526.1
```

```
var(palu$t_cta)
```

```
## [1] 1012134025
```

```
### Poisson segmented regression
poi <- glm(t_cta ~ time + level + trend, data=palu, family = "poisson")
pois <- glm(t_cta ~ time + level + trend + harmonic(time, 3, 12), data=palu, family = "poisson")
summary(poi); summary(pois)
```

```
##
## Call:
## glm(formula = t_cta ~ time + level + trend, family = "poisson",
##     data = palu)
##
## Coefficients:
##              Estimate Std. Error z value Pr(>|z|)
## (Intercept) 11.478478   0.001669 6877.01   <2e-16 ***
## time         0.020787   0.000177  117.44   <2e-16 ***
## level       -0.493790   0.002057 -240.08   <2e-16 ***
## trend       -0.002509   0.000211  -11.89   <2e-16 ***
## ---
## Signif. codes:  0 '***' 0.001 '**' 0.01 '*' 0.05 '.' 0.1 ' ' 1
##
## (Dispersion parameter for poisson family taken to be 1)
##
```


Konu YR, et al. BMJ Glob Health 2024; 9:e013679. doi: 10.1136/bmjgh-2023-013679

```
##     Null deviance: 335729  on 35  degrees of freedom
## Residual deviance: 276794  on 32  degrees of freedom
## AIC: 277283
##
## Number of Fisher Scoring iterations: 4
```

```
##
## Call:
## glm(formula = t_cta ~ time + level + trend + harmonic(time, 3,
##     12), family = "poisson", data = palu)
##
## Coefficients:
##                          Estimate Std. Error  z value Pr(>|z|)
## (Intercept)            11.5720181  0.0018643 6207.262   <2e-16 ***
## time                    0.0112332  0.0002046   54.893   <2e-16 ***
## level                  -0.4172060  0.0022705 -183.752   <2e-16 ***
## trend                  -0.0018925  0.0002276   -8.315   <2e-16 ***
## harmonic(time, 3, 12)1 -0.3715609  0.0007979 -465.678   <2e-16 ***
## harmonic(time, 3, 12)2  0.0619558  0.0007605   81.463   <2e-16 ***
## harmonic(time, 3, 12)3 -0.0296070  0.0007433  -39.832   <2e-16 ***
## harmonic(time, 3, 12)4  0.0301442  0.0007810   38.596   <2e-16 ***
## harmonic(time, 3, 12)5  0.1058961  0.0007496  141.279   <2e-16 ***
## harmonic(time, 3, 12)6 -0.0142881  0.0007434  -19.220   <2e-16 ***
## ---
## Signif. codes:  0 '***' 0.001 '**' 0.01 '*' 0.05 '.' 0.1 ' ' 1
##
## (Dispersion parameter for poisson family taken to be 1)
##
##     Null deviance: 335729  on 35  degrees of freedom
## Residual deviance:  31629  on 26  degrees of freedom
## AIC: 32130
##
## Number of Fisher Scoring iterations: 4
```

```
anova(poi, pois, test = "Chisq")
```

```
## Analysis of Deviance Table
##
## Model 1: t_cta ~ time + level + trend
## Model 2: t_cta ~ time + level + trend + harmonic(time, 3, 12)
##   Resid. Df Resid. Dev Df Deviance  Pr(>Chi)
## 1        32     276794
## 2        26      31629  6   245165 < 2.2e-16 ***
## ---
## Signif. codes:  0 '***' 0.001 '**' 0.01 '*' 0.05 '.' 0.1 ' ' 1
```

```
#### Checking for correlation, autocorrelation and seasonality in the residuals
res_poi <- residuals(pois, type="deviance")
ggtsdisplay(res_poi)
```

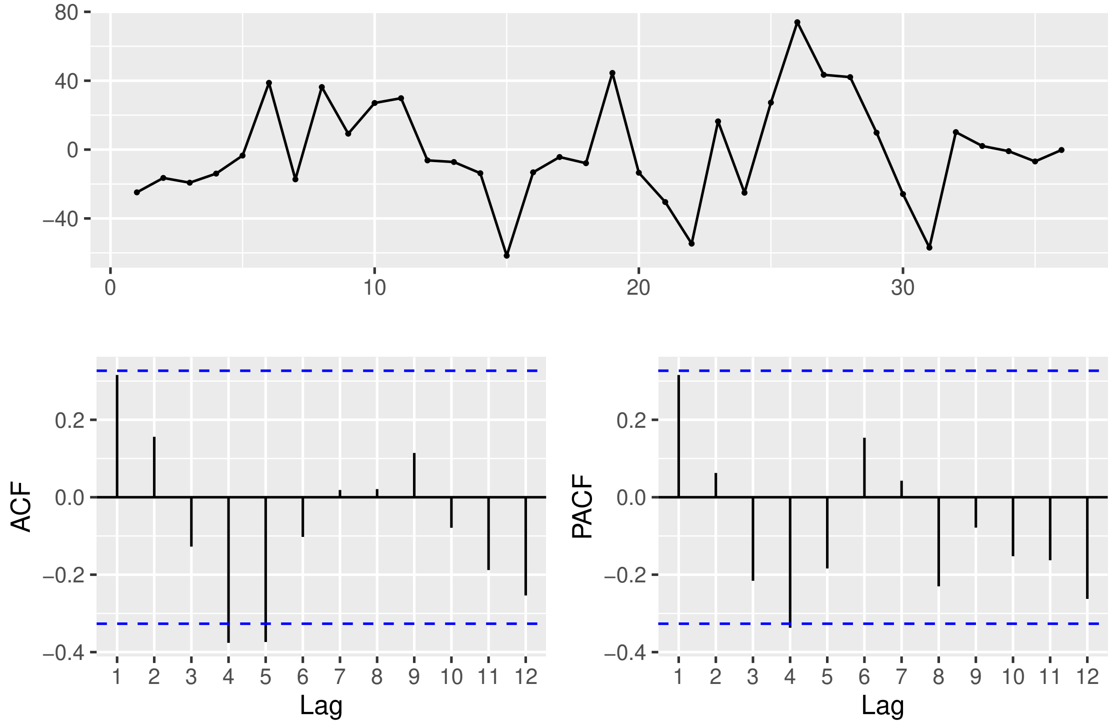


```
!is.null(tbats(ts(res_poi, frequency=12))$seasonal) #No Seasonality in the residuals
```

```
## [1] FALSE
```

```
### Goodness of fit
with(pois, cbind(res.deviance = deviance, df = df.residual, rapport = deviance/df.residual))
```

```
##      res.deviance df  rapport
## [1,]     31628.65 26 1216.487
```

```
with(pois, cbind(res.deviance = deviance, df = df.residual, p = pchisq(deviance, df.residual, lower.tail=FALSE)
```

```
##      res.deviance df p
## [1,]     31628.65 26 0
```

Number of in-patient malaria cases Togo

```
### E(Y)=Var(Y)=Lamda assumption verification
mean(palu$t_hospi)
```

```
## [1] 3135
```

```r
var(palu$t_hospi)
```

```
## [1] 1166695
```

```r
### Poisson segmented regression
pois <- glm(t_hospi ~ time + level + trend + harmonic(time, 3, 12), data=palu, family = "poisson")
poi <- glm(t_hospi ~ time + level + trend, data=palu, family = "poisson")
summary(poi)
```

```
##
## Call:
## glm(formula = t_hospi ~ time + level + trend, family = "poisson",
##     data = palu)
##
## Coefficients:
##              Estimate Std. Error z value Pr(>|z|)
## (Intercept)  7.790585   0.010416  747.94  < 2e-16 ***
## time         0.026478   0.001094   24.20  < 2e-16 ***
## level       -0.309439   0.011942  -25.91  < 2e-16 ***
## trend       -0.008759   0.001266   -6.92 4.52e-12 ***
## ---
## Signif. codes:  0 '***' 0.001 '**' 0.01 '*' 0.05 '.' 0.1 ' ' 1
##
## (Dispersion parameter for poisson family taken to be 1)
##
##     Null deviance: 13427  on 35  degrees of freedom
## Residual deviance: 11928  on 32  degrees of freedom
## AIC: 12290
##
## Number of Fisher Scoring iterations: 4
```

```r
#### Checking for correlation, autocorrelation and seasonality in the residuals
res_poi <- residuals(pois, type="deviance")
ggtsdisplay(res_poi)
```


Konu YR, et al. BMJ Glob Health 2024; 9:e013679. doi: 10.1136/bmjgh-2023-013679

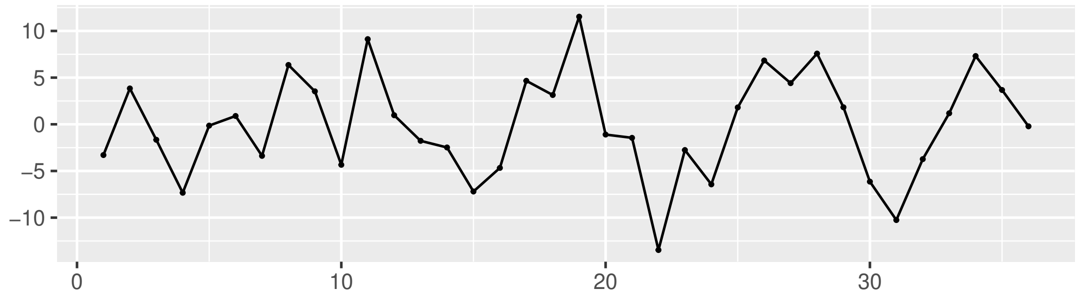

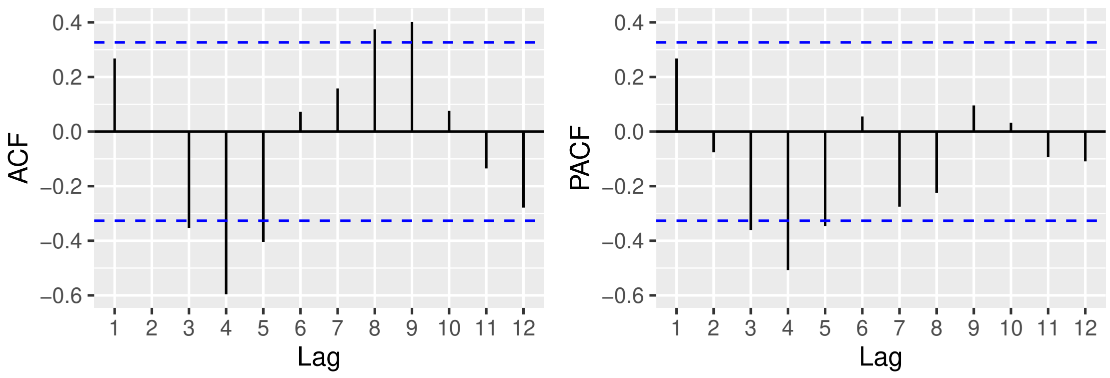


```
!is.null(tbats(ts(res_poi, frequency=12))$seasonal) #No Seasonality in the residuals
```

```
## [1] FALSE
```

```
### Goodness of fit
with(pois, cbind(res.deviance = deviance, df = df.residual, rapport = deviance/df.residual))
```

```
##      res.deviance df  rapport
## [1,]     1086.814 26 41.80053
```

```
with(pois, cbind(res.deviance = deviance, df = df.residual, p = pchisq(deviance, df.residual, lower.tail=FALSE)
```

```
##      res.deviance df            p
## [1,]     1086.814 26 1.41979e-212
```

HIV indicators

ART initiation Togo

```
### E(Y)=Var(Y)=Lamda assumption verification
mean(vih$t_new_arv)
```


Konu YR, et al. BMJ Glob Health 2024; 9:e013679. doi: 10.1136/bmjgh-2023-013679

```
## [1] 1072.917
```

```r
var(vih$t_new_arv)
```

```
## [1] 100156.4
```

```r
### Poisson segmented regression
poi <- glm(t_new_arv ~ time + level + trend, data=vih, family = "poisson")
pois <- glm(t_new_arv ~ time + level + trend + harmonic(time, 3, 12), data=vih, family = "poisson")
summary(poi)
```

```
##
## Call:
## glm(formula = t_new_arv ~ time + level + trend, family = "poisson",
##     data = vih)
##
## Coefficients:
##              Estimate Std. Error z value Pr(>|z|)
## (Intercept)  6.290907   0.019918 315.840  < 2e-16 ***
## time         0.069738   0.001955  35.667  < 2e-16 ***
## level       -0.052700   0.018808  -2.802  0.00508 **
## trend       -0.093393   0.002232 -41.844  < 2e-16 ***
## ---
## Signif. codes:  0 '***' 0.001 '**' 0.01 '*' 0.05 '.' 0.1 ' ' 1
##
## (Dispersion parameter for poisson family taken to be 1)
##
##     Null deviance: 3178.6  on 35  degrees of freedom
## Residual deviance: 1203.5  on 32  degrees of freedom
## AIC: 1527.4
##
## Number of Fisher Scoring iterations: 4
```

```r
summary(pois)
```

```
##
## Call:
## glm(formula = t_new_arv ~ time + level + trend + harmonic(time,
##     3, 12), family = "poisson", data = vih)
##
## Coefficients:
##                         Estimate Std. Error z value Pr(>|z|)
## (Intercept)             6.267216   0.020521 305.409  < 2e-16 ***
## time                    0.072592   0.002068  35.106  < 2e-16 ***
## level                  -0.086263   0.021158  -4.077 4.56e-05 ***
## trend                  -0.095608   0.002280 -41.931  < 2e-16 ***
## harmonic(time, 3, 12)1 -0.022038   0.007546  -2.920   0.0035 **
## harmonic(time, 3, 12)2  0.071609   0.007334   9.764  < 2e-16 ***
## harmonic(time, 3, 12)3 -0.030809   0.007303  -4.219 2.46e-05 ***
## harmonic(time, 3, 12)4 -0.043689   0.007772  -5.621 1.89e-08 ***
## harmonic(time, 3, 12)5  0.089541   0.007235  12.376  < 2e-16 ***
## harmonic(time, 3, 12)6 -0.021966   0.007237  -3.035   0.0024 **
```


Konu YR, *et al. BMJ Glob Health* 2024; 9:e013679. doi: 10.1136/bmjgh-2023-013679

```
## ---
## Signif. codes:  0 '***' 0.001 '**' 0.01 '*' 0.05 '.' 0.1 ' ' 1
##
## (Dispersion parameter for poisson family taken to be 1)
##
##     Null deviance: 3178.56  on 35  degrees of freedom
## Residual deviance:  867.72  on 26  degrees of freedom
## AIC: 1203.6
##
## Number of Fisher Scoring iterations: 4
```

```
anova(poi, pois, test = "Chisq")
```

```
## Analysis of Deviance Table
##
## Model 1: t_new_arv ~ time + level + trend
## Model 2: t_new_arv ~ time + level + trend + harmonic(time, 3, 12)
##   Resid. Df Resid. Dev Df Deviance  Pr(>Chi)
## 1        32    1203.52
## 2        26     867.72  6    335.8 < 2.2e-16 ***
## ---
## Signif. codes:  0 '***' 0.001 '**' 0.01 '*' 0.05 '.' 0.1 ' ' 1
```

```
#### Checking for correlation, autocorrelation and seasonality in the residuals
res_poi <- residuals(pois, type="deviance")
ggtsdisplay(res_poi)
```

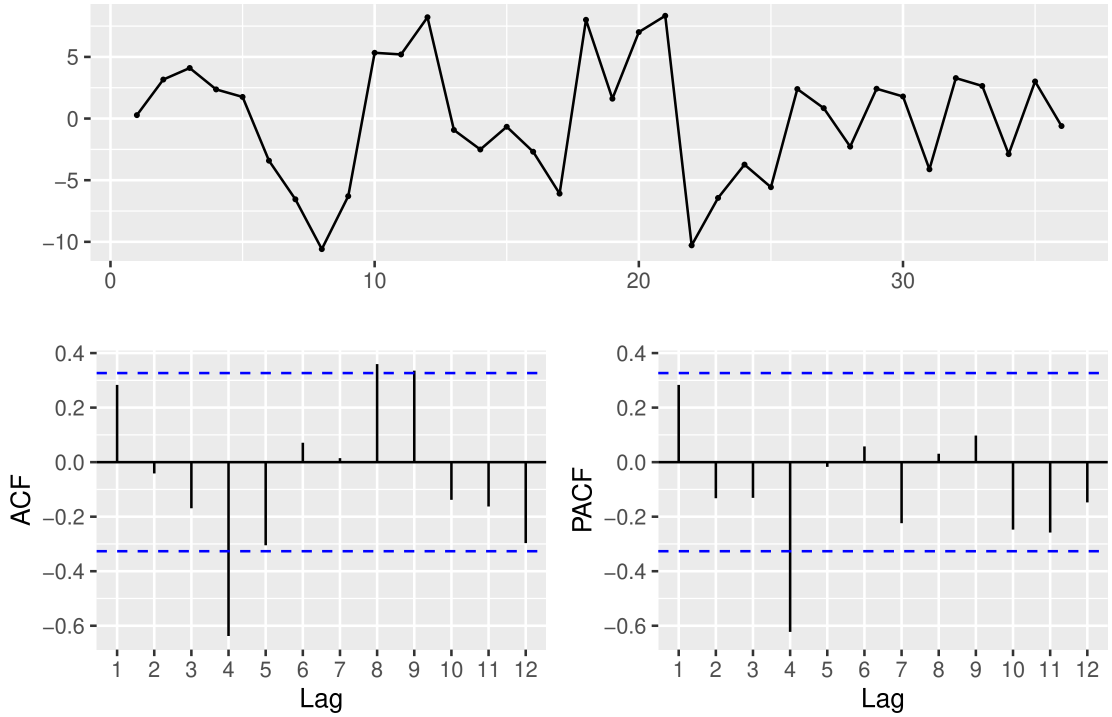

Konu YR, *et al. BMJ Glob Health* 2024; 9:e013679. doi: 10.1136/bmjgh-2023-013679

```r
!is.null(tbats(ts(res_poi, frequency=12))$seasonal) #No Seasonality in the residuals
```

```
## [1] FALSE
```

```r
### Goodness of fit
with(pois, cbind(res.deviance = deviance, df = df.residual, rapport = deviance/df.residual))
```

```
##      res.deviance df  rapport
## [1,]     867.7196 26 33.37383
```

```r
with(pois, cbind(res.deviance = deviance, df = df.residual, p = pchisq(deviance, df.residual, lower.tail=FALSE)
```

```
##      res.deviance df             p
## [1,]     867.7196 26 3.606545e-166
```

Number of infants born to seropositive mothers who received PCR at 1 to 6 weeks of age (with results)

```r
### E(Y)=Var(Y)=Lamda assumption verification
mean(vih$t_pcr)
```

```
## [1] 182.3333
```

```r
var(vih$t_pcr)
```

```
## [1] 1473.714
```

```r
### Poisson segmented regression
poi <- glm(t_pcr ~ time + level + trend, data=vih, family = "poisson")
pois <- glm(t_pcr ~ time + level + trend + harmonic(time, 3, 12), data=vih, family = "poisson")
summary(poi)
```

```
##
## Call:
## glm(formula = t_pcr ~ time + level + trend, family = "poisson",
##     data = vih)
##
## Coefficients:
##              Estimate Std. Error z value Pr(>|z|)
## (Intercept)  5.402601   0.037739 143.156  < 2e-16 ***
## time        -0.015766   0.004273  -3.689 0.000225 ***
## level       -0.182549   0.050945  -3.583 0.000339 ***
## trend        0.030555   0.005082   6.012 1.83e-09 ***
## ---
## Signif. codes:  0 '***' 0.001 '**' 0.01 '*' 0.05 '.' 0.1 ' ' 1
##
## (Dispersion parameter for poisson family taken to be 1)
```


Konu YR, *et al. BMJ Glob Health* 2024; 9:e013679. doi: 10.1136/bmjgh-2023-013679

```
##
##     Null deviance: 287.00  on 35  degrees of freedom
## Residual deviance: 217.71  on 32  degrees of freedom
## AIC: 478.5
##
## Number of Fisher Scoring iterations: 4
```

```
summary(pois)
```

```
##
## Call:
## glm(formula = t_pcr ~ time + level + trend + harmonic(time, 3,
##     12), family = "poisson", data = vih)
##
## Coefficients:
##                          Estimate Std. Error z value Pr(>|z|)
## (Intercept)              5.395726   0.041275 130.727  < 2e-16 ***
## time                    -0.011753   0.004733  -2.483   0.0130 *
## level                   -0.307468   0.055511  -5.539 3.04e-08 ***
## trend                    0.029948   0.005318   5.631 1.79e-08 ***
## harmonic(time, 3, 12)1 -0.107773   0.018523  -5.818 5.95e-09 ***
## harmonic(time, 3, 12)2 -0.003019   0.017776  -0.170   0.8652
## harmonic(time, 3, 12)3 -0.001538   0.017668  -0.087   0.9306
## harmonic(time, 3, 12)4 -0.163220   0.018460  -8.842  < 2e-16 ***
## harmonic(time, 3, 12)5  0.037646   0.017616   2.137   0.0326 *
## harmonic(time, 3, 12)6  0.020880   0.017618   1.185   0.2359
## ---
## Signif. codes:  0 '***' 0.001 '**' 0.01 '*' 0.05 '.' 0.1 ' ' 1
##
## (Dispersion parameter for poisson family taken to be 1)
##
##     Null deviance: 287.000  on 35  degrees of freedom
## Residual deviance:  92.457  on 26  degrees of freedom
## AIC: 365.25
##
## Number of Fisher Scoring iterations: 4
```

```
anova(poi, pois, test="Chisq")
```

```
## Analysis of Deviance Table
##
## Model 1: t_pcr ~ time + level + trend
## Model 2: t_pcr ~ time + level + trend + harmonic(time, 3, 12)
##   Resid. Df Resid. Dev Df Deviance  Pr(>Chi)
## 1        32    217.706
## 2        26     92.457  6   125.25 < 2.2e-16 ***
## ---
## Signif. codes:  0 '***' 0.001 '**' 0.01 '*' 0.05 '.' 0.1 ' ' 1
```

```
#### Checking for correlation, autocorrelation and seasonality in the residuals
res_poi <- residuals(pois, type="deviance")
ggtsdisplay(res_poi)
```

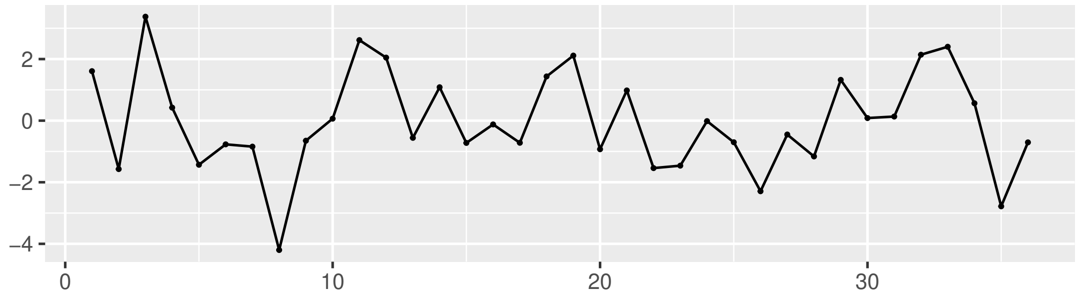

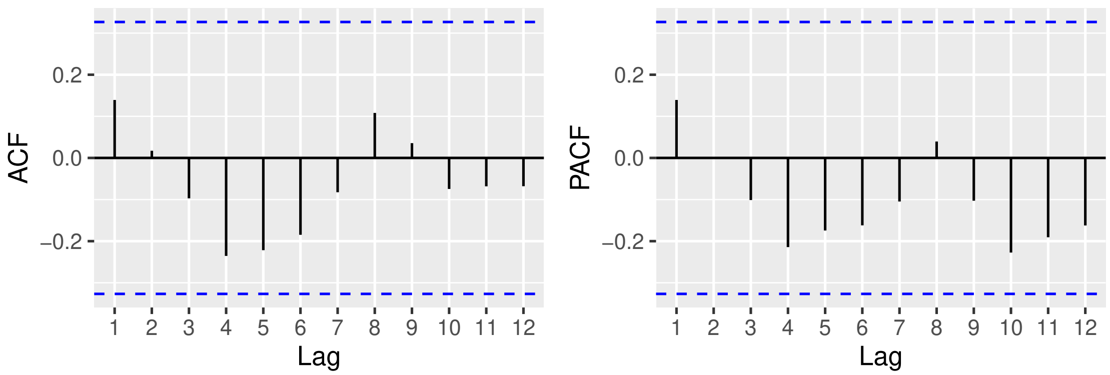


```
!is.null(tbats(ts(res_poi, frequency=12))$seasonal) #No Seasonality in the residuals
```

```
## [1] FALSE
```

```
### Goodness of fit
with(pois, cbind(res.deviance = deviance, df = df.residual, rapport = deviance/df.residual))
```

```
##      res.deviance df  rapport
## [1,]     92.45688 26 3.556034
```

```
with(pois, cbind(res.deviance = deviance, df = df.residual, p = pchisq(deviance, df.residual, lower.tail=FALSE)
```

```
##      res.deviance df            p
## [1,]     92.45688 26 2.229567e-09
```

Number of people tested for HIV

```
### E(Y)=Var(Y)=Lamda assumption verification
mean(vih$t_test)
```

```
## [1] 40922.92
```


Konu YR, *et al. BMJ Glob Health* 2024; 9:e013679. doi: 10.1136/bmjgh-2023-013679

```
var(vih$t_test)
```

```
## [1] 23826067
```

```
### Poisson segmented regression
poi <- glm(t_test ~ time + level + trend, data=vih, family = "poisson")
pois <- glm(t_test ~ time + level + trend + harmonic(time, 3, 12), data=vih, family = "poisson")
summary(poi)
```

```
##
## Call:
## glm(formula = t_test ~ time + level + trend, family = "poisson",
##     data = vih)
##
## Coefficients:
##               Estimate Std. Error z value Pr(>|z|)
## (Intercept) 10.5871383  0.0027596 3836.44   <2e-16 ***
## time        -0.0049835  0.0003063  -16.27   <2e-16 ***
## level        0.0542299  0.0033866   16.01   <2e-16 ***
## trend        0.0140486  0.0003523   39.88   <2e-16 ***
## ---
## Signif. codes:  0 '***' 0.001 '**' 0.01 '*' 0.05 '.' 0.1 ' ' 1
##
## (Dispersion parameter for poisson family taken to be 1)
##
##     Null deviance: 20350  on 35  degrees of freedom
## Residual deviance: 12262  on 32  degrees of freedom
## AIC: 12719
##
## Number of Fisher Scoring iterations: 4
```

```
summary(pois)
```

```
##
## Call:
## glm(formula = t_test ~ time + level + trend + harmonic(time,
##     3, 12), family = "poisson", data = vih)
##
## Coefficients:
##                          Estimate Std. Error  z value Pr(>|z|)
## (Intercept)            10.5719576  0.0028314 3733.836  < 2e-16 ***
## time                   -0.0041804  0.0003132  -13.349  < 2e-16 ***
## level                   0.0597852  0.0035984   16.614  < 2e-16 ***
## trend                   0.0132640  0.0003513   37.754  < 2e-16 ***
## harmonic(time, 3, 12)1  0.0123872  0.0012341   10.038  < 2e-16 ***
## harmonic(time, 3, 12)2  0.0691855  0.0011799   58.635  < 2e-16 ***
## harmonic(time, 3, 12)3  0.0003636  0.0011815    0.308 0.758261
## harmonic(time, 3, 12)4  0.0037054  0.0012153    3.049 0.002297 **
## harmonic(time, 3, 12)5  0.0595717  0.0011698   50.926  < 2e-16 ***
## harmonic(time, 3, 12)6 -0.0044643  0.0011683   -3.821 0.000133 ***
## ---
## Signif. codes:  0 '***' 0.001 '**' 0.01 '*' 0.05 '.' 0.1 ' ' 1
```


Konu YR, *et al. BMJ Glob Health* 2024; 9:e013679. doi: 10.1136/bmjgh-2023-013679

```
##
## (Dispersion parameter for poisson family taken to be 1)
##
##     Null deviance: 20350.1  on 35  degrees of freedom
## Residual deviance:  6055.5  on 26  degrees of freedom
## AIC: 6523.7
##
## Number of Fisher Scoring iterations: 3
```

```
anova(poi, pois, test = "Chisq")
```

```
## Analysis of Deviance Table
##
## Model 1: t_test ~ time + level + trend
## Model 2: t_test ~ time + level + trend + harmonic(time, 3, 12)
##   Resid. Df Resid. Dev Df Deviance  Pr(>Chi)
## 1        32    12262.4
## 2        26     6055.5  6   6206.9 < 2.2e-16 ***
## ---
## Signif. codes:  0 '***' 0.001 '**' 0.01 '*' 0.05 '.' 0.1 ' ' 1
```

```
#### Checking for correlation, autocorrelation and seasonality in the residuals
res_poi <- residuals(pois, type="deviance")
ggtsdisplay(res_poi)
```

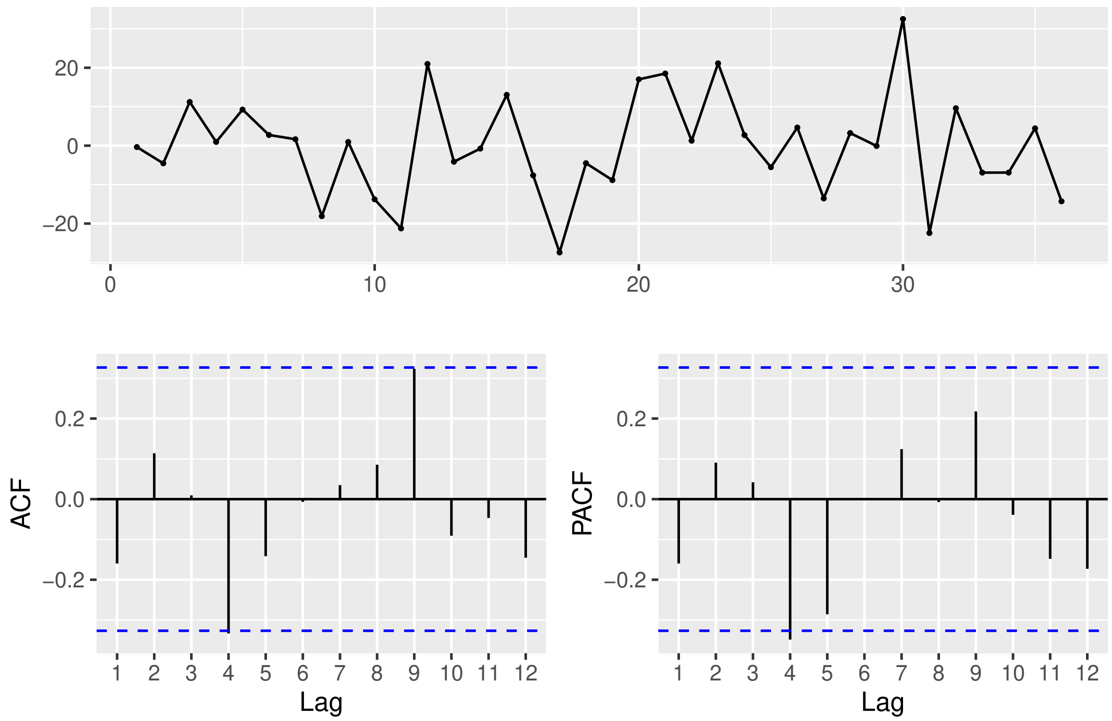

Konu YR, *et al. BMJ Glob Health* 2024; 9:e013679. doi: 10.1136/bmjgh-2023-013679

```
!is.null(tbats(ts(res_poi, frequency=12))$seasonal) #No Seasonality in the residuals
```

```
## [1] FALSE
```

```
### Goodness of fit
with(pois, cbind(res.deviance = deviance, df = df.residual, rapport = deviance/df.residual))
```

```
##      res.deviance df  rapport
## [1,]     6055.479 26 232.9031
```
